# Evaluation of water from Lake Coatetelco in central-south Mexico and surrounding groundwater wells for drinking and irrigation, and the possible health risks

**DOI:** 10.1007/s11356-023-30488-7

**Published:** 2023-10-26

**Authors:** Priyadarsi D. Roy, Oscar Agesandro García-Arriola, Sekar Selvam, Irma Gabriela Vargas-Martínez, José Luis Sánchez-Zavala

**Affiliations:** 1 Instituto de Geología, Universidad Nacional Autónoma de México, Del. Coyoacán, 04510 Ciudad de Mexico, Mexico; 2 Posgrado en Ciencias del Mar y Limnología, Universidad Nacional Autónoma de México, Del. Coyoacán, 04510 Ciudad de Mexico, Mexico; 3 Department of Geology, V.O. Chidambaram College, Tuticorin, Tamil Nadu 628008 India; 4 Carrera de Ingeniería Geológica, Facultad de Ingeniería, Universidad Nacional Autónoma de México, Del. Coyoacán, 04510 Ciudad de Mexico, Mexico

**Keywords:** Hydrochemistry, Fluoride and nitrate enrichment, Water quality assessment, Irrigation indices, Hazard quotient, Mexico

## Abstract

Due to an increasing reduction of hydrological resources across Mexico and their growing contamination from global warming and anthropogenic activities, this study evaluated water from the perennial Lake Coatetelco (Ca–Mg–HCO_3_) in tropical central-southern Mexico and groundwater (Ca–Mg–HCO_3_ and Na–HCO_3_–Cl) from the surrounding wells for drinking as well as irrigation qualities. Comparison with the WHO guidelines and the estimated water quality indices (DWQI and IWQI) grouped almost all the samples collected after the warm season rainfall in excellent and good categories (DWQI < 100) for drinking, even though fluoride remained > 1.5 mg/L in 50% samples. Except for one groundwater sample, all showed > 25% permeability (classes I and II) in Donnen classification indicating their suitability for irrigation. USSL and Wilcox classifications, however, catalogued some in the high-salinity hazard group and some as doubtful for irrigating regular plants. Samples from about 53% wells were also in high and severe restriction categories of IWQI for the irrigation. Total Hazard Quotient Index (THQI) for estimating the non-carcinogenic risk (HQ_fluoride_ > 1) showed that at least one lake water sample and 53% of groundwater might expose the adult and child population to dental and skeletal fluorosis. This water quality assessment data posterior to the rainfall season could be useful as a baseline for both the short- and long-term monitoring in attention to the United Nation’s Sustainable Development Goal 6.

## Introduction

Groundwater is the main resource of potable water across the globe as well as of the water used in irrigated agriculture and industrial activity (e.g., Adimalla 2019, 2020; Alarcón-Herrera et al. 2020; Subba Rao et al. 2020). Almost half of the global drinking water and approximately 40% of irrigation water come from the aquifers, and around one-third of the overall population suffers from water shortage every year (Mekonnen and Hoekstra 2016; Abascal et al. 2022). The population growth, aridification caused from the global warming, and the enhancement in industrial activities, including the agriculture, have either polluted or degraded the available water resources by increasing the levels of contaminants (both geogenic and anthropogenic), and thus, augmented the public health threat from this natural resource (e.g., Kimambo et al. 2019; Alarcón-Herrera et al. 2020; Abascal et al. 2022). About 200 million people in more than 25 countries are presently exposed to the risk of dental and skeletal fluorosis, bone fractures, kidney stones, low birth rate and thyroid function, and glucose tolerance as well as low IQ levels from drinking the water with F > 1.5 mg/L (WHO 2017; Duan et al. 2018; Kimambo et al. 2019). Similarly, the exposure to extreme high (> 50 mg/L) nitrate for a longer period leads to adverse health risk, such as methemoglobinaemia, thyroid or cancer, hypertension, diabetes, spontaneous abortion, respiratory tract infection, and change in the immune system (Gupta et al. 2000; Fewtrell 2004; Martínez et al. 2017; Tokazhanov et al. 2020). The interaction with fluoride-rich minerals such as fluorite, mica, amphibole, villiaumite, and topaz present in aquifer lithologies as well as the chemical weathering of volcanic rocks leads to F enrichment in the water bodies (Cronin et al. 2000; Yadav et al. 2021; Alarcón-Herrera et al. 2020). Higher nitrate, however, is a direct consequence of fertilizers as about 60% of water bodies with elevated nitrate occur near the croplands (Singh and Craswell 2021). Hence, the objective of United Nation’s Sustainable Development Goal (SDG) 6 and target 6.4 is to address water scarcity by substantially increasing the water-use efficiency and access to safe water across all sectors by 2030.

Like many other developing countries, Mexico too relies heavily on groundwater for drinking and irrigation, contributing about 39% (i.e., 35, 000 hm^3^/a) of the total volume consumed from its 653 aquifers, including the 115 overexploited ones present in the semi-arid and arid central and northern regions (CONAGUA 2019). The perennial lakes are also an important source of water in the central Mexico. For example, the largest Lake Chapala (> 1000 km^2^) can store up to 8126 hm^3^ of water and smaller lakes like the Lake Tequesquitengo (< 10 km^2^) has a storage volume of up to 160 hm^3^. About 20 million people, including 6.5 million children, in Mexico are exposed to water with F above the permissible limit, and about half of them are from the central and northern parts that presently experience prolonged droughts and have volcanic deposits as well as F-bearing limestone as the aquifer lithologies (Reyes-Gómez et al. 2017; Navarro et al. 2017; Roy et al. 2021). Average fluoride content in groundwater is almost half (0.6 mg/L) in regions of Mexico with an annual precipitation above 1000 mm compared to the regions with < 700 mm of average annual rainfall (1.2 mg/L; Alarcón-Herrera et al. 2020). LaFayette et al. (2020) reported up to 15.5 mg/L of F in groundwater from the Independent Basin of central Mexico with abundant andesite. Similarly, Gutierrez and Alarcón-Herrera (2022) reported F above the permissible limit in 36–52% wells of the central and northern Mexico and fluoride up to 28 mg/L in areas with abundant felsic volcanic rocks. In a semi-arid region of south India (< 1000 mm/a precipitation), Adimalla (2019) reported up to 7.1 mg/L of fluoride and up to 440 mg/L of nitrate with about half of the groundwater samples containing contaminants above the maximum permissible limits of the World Health Organization (WHO). The groundwater, in an important agricultural district of northern Mexico at the Comarca Lagunera, has NO_3_ up to 109 mg/L, and the values above WHO-recommended safe limit of 10 mg/L in 32% wells were mostly from the intensive manure usage and urban sewage (Torres-Martínez et al. 2021). In a previous study of this basin, Calleros et al. (2012) reported a possible health risk for 45% of the 1- to 12-year-old children of this area from methemoglobin, with relatively more threat for male compared to the female population. Fluorosis is more common in Mexico compared to the methemoglobin and the data of national caries survey of 2001 indicated an overall fluorosis prevalence of 27.9% (Betancourt-Lineares et al. 2013). The survey of 2011–2014, however, revealed that the dental fluorosis is increasingly becoming a public health problem not only in the semi-arid and arid regions of central and northern Mexico, but only in the sub-humid to humid central and southern regions. For example, the prevalence level on 12-year-old school-going children in Morelos state increased from 3.2 to 33.7% after a decade with an average 0.28–0.50 teeth/person showing permanent caries (ENCD 2018). This could be due to increase of F in the groundwater of this region. Huízar Álvarez et al. (2016) also have reported up to 1.90 mg/L of F in groundwater and spring water from the Tenextepango area of Morelos state.

Consistent with the global perspectives, the simulations for Mexico indicate significant reduction in average precipitation in different parts from an increase in the global average temperature between 1.5 and 4 °C in the short, middle, and long terms over a century (Arias et al. 2021). Reduced rainfall and warmer conditions, possibly more than the global average, along with the overexploitation would lead to a critical negative annual imbalance and deterioration in the quality and quantity of available water resources. Under different scenarios of emission and adaptation levels, the agricultural productivity in Mexico could decline up to 48% by the end of this century (e.g., Feng et al. 2010). Concurrently, the application of saline water in irrigation would increase the kinematic viscosity and cause low soil permeability. It could also reduce the movement of water from soil to the plant roots, branches, and leaves (e.g., Nagarajan et al. 2010; Zhou et al. 2020). Irrigation with the high alkalinity water generally reduces the soil nutrients (e.g., Sharma et al. 2017). Here, we attempted to evaluate the quality of water from Lake Coatetelco and surrounding groundwater wells located in the central-southern Mexico, with significant agricultural activity, for drinking and irrigation purposes by comparing the physicochemical characteristics with WHO guidelines and estimating the water quality indices such as DWQI and IWQI. The irrigation quality was also assessed using the USSL classification, Wilcox diagram, and Doneen classification. The possible non-carcinogenic health risks from nitrate and fluoride in water samples were evacuated by estimating the hazard quotients for adults (> 21 years) and children (average weight 20 kg).

## Study area

**Fig. 1:**
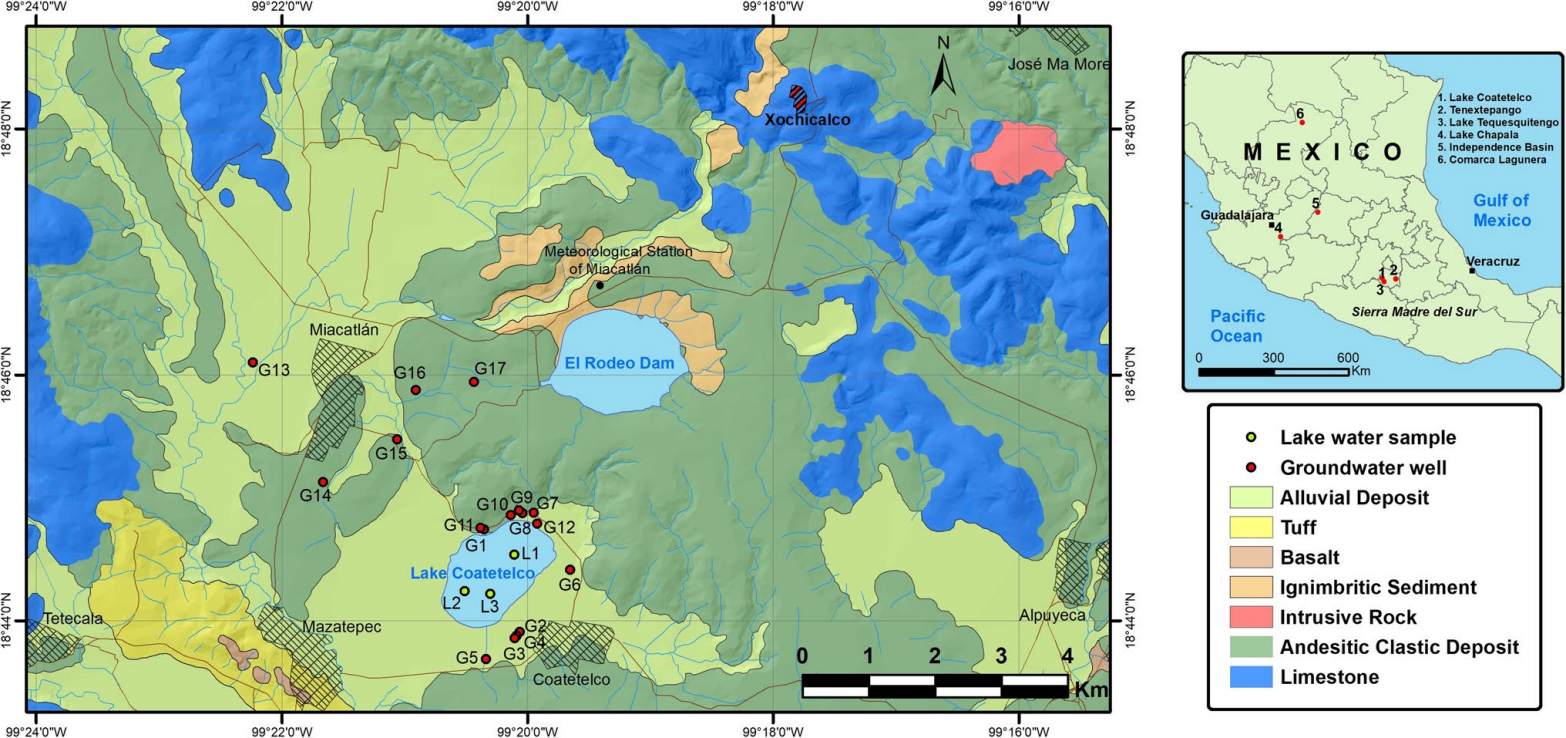
**a** Map showing Lake Coatetelco (Morelos state) in central-south Mexico and some other sites discussed in the paper. **b** Samples of water from the shallow lake (*n* = 3) and groundwater (*n* = 17) from the surrounding wells used for domestic and agricultural usages were collected after the warm rainfall season

Lake Coatetelco is located at central-south Mexico (Morelos state), and physiographically, it belongs to the Sierra Madre del Sur province (Fig. 1). It has a tropical, warm semi-humid climate with the nearest meteorological station of Miacatlán (Fig. 1) registering an average annual precipitation of ca.1080 mm between 1970 and 2016 CE with > 85% of it occurring in the warmer months of May-to-September and the average annual evaporation of about 1940 mm (source: National Meteorological Service of Mexico or SMN). This perennial lake (length: ca. 2 km and width: ca. 1.5 km) has a shallow water column with a mean depth of < 0.5 m. Over-extraction of groundwater and enhanced erosion in the watershed from increasing urbanization in the recent years has reduced the maximum water depth to < 3 m.

The study area belongs to northwestern part of the unconfined, heterogenous, and anisotropic Zacatepec aquifer (total surface area: about 1279 km^2^) comprising of about 150-m-thick unconsolidated Cenozoic alluvial deposits as well as sandstone and conglomerates intercalated with volcanic deposits (basalt and andesite) and ignimbrites in the upper part (CONAGUA 2020; Rivera-Carranza et al. 1998). The lower part of this aquifer is represented by secondary permeability in the underlying Mesozoic limestones (Morelos and Cuautla Formations) caused from fracturing and dissolution (Fries 1960; Rivera-Carranza et al. 1998; CONAGUA 2020). The general static levels of this shallow aquifer vary between 5 and 80 m, and it is, however, < 20 m in the study area. Hydraulic transmissivity (4.82–12.1 × 10^−3^ m^2^/s) and hydraulic conductivity (0.005–0.14 × 10^−3^ m^2^/s) are variable, and it is a surplus aquifer with annual recharge of 85.3 hm^3^ and extraction of 56.4 hm^3^ (CONAGUA 2020). Most of the groundwater extracted is used for agricultural activities (30%, 17 hm^3^) and urban consumption (47.5%, 26.8 hm^3^), and the industrial usage is limited to 1.5% (0.9 hm^3^) of the total extraction (CONAGUA 2020).

The superficial phaeozem soil and vertisol are currently used for agricultural activities such as the farming of lemon, papaya, avocado, mango, guava, and banana, as well as the cultivation of dominantly corn and sugarcane (INEGI 1984; García Flores et al. 2019). About 24% of the population of the study area is marginalized and vulnerable due to social deficiencies such as lack of access to food with the main economic activities restricting to agriculture, livestock, and fishing (García Flores et al. 2019).

## Sampling, analysis, and evaluation method

A total of 3 water samples (L1–L3) from the shallow water column of Lake Coatetelco and groundwater samples from 17 surrounding bore wells (G1–G17) with water depths below 20 m were collected in Teflon bottles immediately after the warmer rainfall season in October 2020 as the objective was to evaluate the physicochemical characteristics of water resources in their dilute states for the drinking and irrigation quality assessments as well as evaluation of the possible health risks from NO_3_ and F consumptions (Fig. 1). The study area receives more than 85% of its annual precipitation between May and September, and hence, the samples collected in October represented the water resources in their most dilute conditions. The quality assurance and quality control (QA/QC) procedures ensured the reliability of water samples. In-field QA/QC measures included collection of samples in duplicates and their preservation in refrigeration using ice cubes and ice box from the sampling sites to the laboratory. All the Teflon bottles were pre-cleaned with water from Milli-Q direct water purification system and rinsed thrice in field with water from the sample bore well and lake water before the sample collection. A pair of Teflon bottles with water from the Milli-Q system were used as blanks during the entire expedition and they were measured in laboratory to detect possible contamination which occurred in the field. After measuring the electrical conductivity (EC), total dissolved solids (TDS), and hydrogen ion concentration (pH) with a portable Hanna instrument (HI 98130) in the field, all the samples were transported under 4 °C to the laboratory. The cations (Na^+^, Ca^2+^, Mg^2+^, and K^+^) and anions (HCO_3_^−^, Cl^−^, SO_4_^2−^, and NO_3_^2−^) were analyzed using a Waters liquid chromatograph comprising of a binary pump (Model 1525), auto sampler (Model 717 plus), and conductivity detector (Model 432) after Zamora-Martínez et al. (2016). This ion exchange chromatography has separate ion exchange columns for anions (IC-PAK TM Anion HR, 46 mm × 75 mm, Waters) and cations (Metrosep C6-250/4.0, Metrohm). All samples were filtered through 0.45-micron pore size nylon membranes prior to the analysis and the autosampler was rinsed with type I water to avoid cross contamination between each injection. An ion-selective electrode (HANAA instruments) combined with a PC 700 (Oakton) benchtop pH/conductivity meter analyzed F^−^ concentrations. The quality parameters were controlled by using six different NIST-certified high purity standard reference materials (i.e., IC-4–100, IC-1-A-100, IC-1–3-100, IC-1-A-100, etc.) and using the methods certified under the Mexicana and International norms (ISO-9001 and ISO-17025). Duplicate samples were analyzed after every 10 samples, and the accuracy of chemical parameters was evaluated with the calculation of expanded uncertainty at 95% confidence level and the Ionic Balance Error (IBE = ΣCation − ΣCation/ΣCation + ΣAnion × 100).

### Drinking water quality evaluation

Comparison of major ion compositions with World Health Organization guidelines (WHO 2017) was the first-hand filter to evaluate the drinking suitability. Additionally, we evaluated overall drinking water quality index (DWQI) by assessing the similarities of physicochemical parameters with WHO limits through a mathematical computation and assigning different weights (wi) and relative weights (Wi) based on their influences considered from Yi et al. (2019), Marghade (2020), Verma et al. (2020), and Wu et al. (2020). Critical water quality parameters such as TDS, Cl, SO_4_, NO_3_, and F were allotted the maximum weight of 5, and HCO_3_ was assigned the minimum weight of 1 (e.g., Vasanthavigar et al. 2010; Tiwari et al. 2017). Other parameters such as pH (3), Ca (3), Mg (3), Na (4), and K (2) were assigned weights varying between 2 and 4 (Verma et al. 2020).

The relative weight (W_i_) of each parameter (pH: 0.073; TDS: 0.122; HCO_3_: 0.024; Cl: 0.122; SO_4_: 0.122; NO_3_: 0.122; F: 0.122; Ca: 0.073; Mg: 0.073; Na: 0.098; K: 0.049) was calculated using the following equation:

$$\mathrm{Wi}= \frac{{\mathrm{w}}_{\mathrm{i}}}{{\sum }_{\mathrm{i}=1}^{\mathrm{n}}{\mathrm{w}}_{\mathrm{i}}}$$

where w_i_ is the weight of each parameter and n is the number of parameters. A quality rating scale (q_i_) for each parameter was calculated by dividing the concentration in each water sample with relative standard concentration recommended by the WHO (2017) guidelines and multiplied by 100:

$$\mathrm{qi}= \frac{({\mathrm{C}}_{\mathrm{i}})}{({\mathrm{S}}_{\mathrm{i}})}\times 100$$

where qi is the quality rating scale, C_i_ is concentration of each chemical parameter in each water sample in mg/L, and S_i_ is the standard for each chemical parameter in mg/L according to the WHO (2017) guidelines.

Finally, the DWQI was estimated using the value of SI_*i*_ (= w_*i*_ × q_*i*_) in the following equation:

$$\mathrm{DWQI}=\sum_{i=1}^{n}{\mathrm{SI}}_{i}$$

where SI_i_ is the sub-index of i^th^ parameter, q_i_ is the rating based on concentration of i^th^ parameter, and n is the number of parameters. It switched the water quality parameters of all the lake and groundwater samples into codes to assess their suitability for drinking by separating them into five different categories from excellent (DWQI > 50) to unsuitable (DWQI > 300).

### Irrigation water quality evaluation

Multiple indices such as total hardness (TH), salinity hazard, sodium adsorption ratio (SAR), residual sodium carbonate (RSC), sodium percentage (Na%), Kelley ratio (KR), magnesium ratio (MR), potential salinity (PS), and permeability index (PI) evaluated the irrigation water quality of both the lake and groundwater samples (e.g., Richards 1954; Doneen 1962, 1964; Kelley 1963; Ragunath 1987). Additionally, the overall irrigation quality was also evaluated by calculating the irrigation water quality index (IWQI; e.g., Rana et al. 2018; Sharma et al. 2020).

Total hardness (TH) index determined accumulation of divalent calcium and magnesium ions (in meq/L) in water samples by using the equation of Ragunath (1987):

$$\mathrm{TH}=\left(\mathrm{Ca}+\mathrm{Mg}\right)\times 50$$

Salinity hazard considered the values of EC for classifying the sample into five different categories between excellent (EC < 250 µS/cm) and unsuitable (EC > 3000 µS/cm). Sodium adsorption ratio (SAR) considered the concentrations of Na with respect to Ca and Mg (in meq/L) as per Richards (1954):

$$\mathrm{SAR}= \frac{{\mathrm{Na}}^{+}}{\sqrt{\frac{{\mathrm{Ca}}^{2+}+{\mathrm{Mg}}^{2+}}{2}}}$$

Residual sodium carbonate (RSC) evaluated the water quality by considering the concentrations of carbonate and bicarbonate ions against the proportions of calcium and magnesium ions (in meq/L) in the formula of Eaton (1950):

$$\mathrm{RSC}=\left({\mathrm{CO}}_{3}+{\mathrm{HCO}}_{3}\right)-(\mathrm{Ca}+\mathrm{Mg})$$

The index of Na percentage (Na %) determined irrigation water quality from concentrations (meq/L) of all dissolved cations in the formula of Doneen (1962):

$${\mathrm{Na}}^{+}\mathrm{\%}=\frac{{\mathrm{Na}}^{+}+{\mathrm{K}}^{+}}{{\mathrm{Ca}}^{2+}+{\mathrm{Mg}}^{2+}+{\mathrm{Na}}^{+}+{\mathrm{K}}^{+}}\times 100$$

Kelley ratio (KR) evaluated the ratio of concentrations of Na against Ca and Mg (in meq/L) using the formula of Kelley (1963):

$$\mathrm{KR}=\frac{{\mathrm{Na}}^{+}}{{\mathrm{Ca}}^{2+}+{\mathrm{Mg}}^{2+}}$$

Magnesium ratio (MR) represented the concentrations of Mg against the contents of both Ca and Mg (in meq/L) as per Paliwal (1972):

$$\mathrm{MR}=\frac{{\mathrm{Mg}}^{2+}}{{\mathrm{Ca}}^{2+}+{\mathrm{Mg}}^{2+}}\times 100$$

The potential salinity (PS) index considered chloride ion concentration and half of sulfate concentration in meq/L as per Doneen (1964):

$$\mathrm{PS}= \mathrm{Cl}+ \frac{{\mathrm{SO}}_{4}}{2}$$

Finally, the permeability index (PI) assessed suitability of water for irrigation by evaluating water flux efficiency (permeability) in soil using the concentrations of Na, Ca, Mg, and HCO_3_ in meq/L by following Doneen (1964):

$$\mathrm{PI}= \frac{(\mathrm{Na }+ \sqrt{{\mathrm{HCO}}_{3}})}{(\mathrm{Ca}+\mathrm{Mg}+\mathrm{Na})} \times 100$$

All these indices separated the water samples into different categories such as safe and unsafe or suitable or unsuitable classes. IWQI determined the overall suitability of water quality for irrigation as follows (e.g., Rana et al. 2018; Sharma et al. 2020):

$$IWQI=\sum_{i=1}^{n}{q}_{i}{w}_{i}$$

where *w*_*i*_ represents normalized weight of the i^th^ parameter (EC: 0.211; Na: 0.204; HCO_3_: 0.202; Cl: 0.194; SAR: 0.189) and *q*_*i*_ represents the quality of i^th^ parameter (between 0 and 100, Table 1) determined using the following equation:

$${q}_{i}={q}_{max}-\left(\frac{\left[\left({x}_{ij}-{x}_{inf}\right){q}_{iamp}\right]}{{x}_{amp}}\right)$$

where *q*_*max*_ represents the highest value of q_i_ for each class, *x*_*ij*_ is the value of each parameter, *x*_*inf*_ represents the minimum value of the class to which the indicator belongs, *q*_*imap*_ is the class amplitude, and *x*_*amp*_ represents class amplitude in which the properties belong. IWQI values grouped the water samples into five different categories based on their suitability for irrigation with IWQI of 85–100 representing the water samples of no restriction for irrigation and IWQI of 0–40 representing water samples of severe restriction.

**Table 1:** Limitation of parameters used for the quality measurement (q_i_) in IWQI calculations as per Meireles et al. (2010) for the lake and groundwater samples from and around Lake Coatetelco, central-south Mexico

q_i_	EC (dS/m)	Na	Cl	HCO_3_	SAR (meq/L)^1/2^
		meq/L			
85–100	0.2 ≤ EC < 0.75	2 ≤ Na < 3	1 ≤ Cl < 4	1 ≤ HCO_3_ < 1.5	2 ≤ SAR < 3
60–85	0.75 ≤ EC < 1.50	3 ≤ Na < 6	4 ≤ Cl < 7	1.5 ≤ HCO_3_ < 4.5	3 ≤ SAR < 6
35–60	1.50 ≤ EC < 3.00	6 ≤ Na < 12	7 ≤ Cl < 10	4.5 ≤ HCO_3_ < 8.5	6 ≤ SAR < 12
0–35	EC < 0.20 or EC ≥ 3.0	Na < 2 or Na ≥ 12	Cl < 1 or Cl ≥ 10	HCO_3_ < 1 or HCO_3_ ≥ 8.5	SAR < 2 or SAR ≥ 12

### Health risk assessment

The possible adverse health impacts on men, women, and children were assessed following the recommendation of United States Environmental Protection Agency (USEPA 2004). The intake of both NO_3_ and F through drinking was considered here as the main pathway and their non-carcinogenic risks were estimated by calculating the average daily exposure dose (DE) through ingestion of lake and groundwater (mg/kg/day) using the formula of USEPA (2004) like the studies of Adimalla et al. (2018), Karunanidhi et al. (2020a), and Subba Rao et al. (2020):

$$\mathrm{DE}=\frac{{\mathrm{C}}_{\mathrm{P}}\times \mathrm{IR}\times \mathrm{ED}\times \mathrm{EF}}{\mathrm{AB}\times \mathrm{AE}}$$

where C_P_ is pollutant concentration (mg/L), IR is ingestion rate per unit time (adult male and female: 3.229 L/day and child: 1.006 L/day; USEPA analysis 2019 data at 95^th^ percentile (ATSDR 2023)), ED is exposure duration (male: 64; female; 67; children: 12 years; Subba Rao et al. 2020), and EF is exposure frequency (365 days/year; Subba Rao et al. 2020). We considered the average body weight (AB) in the context of Mexico as 74 kg for adult male, 68 kg for adult female, and 20 kg for a child. AE is the average age exposure time (adult male: 23,360; adult female: 24,455; children: 4380 days; Subba Rao et al. 2020).

The hazard quotient (HQ) was applied to evaluate risks from fluoride and nitrate in water samples using the equation:

$$\mathrm{HQ}=\frac{\mathrm{DE}}{\mathrm{RfD}}$$

where HQ is the hazard quotient for nitrate and fluoride, which is a measure of non-carcinogenic chronic hazard, and RfD represents the reference dose for chronic oral exposure (mg/kg/day) to both the pollutants (NO_3_: 1.60; F: 0.06 mg/kg/day; Adimalla 2019; Alarcón-Herrera et al. 2020). Finally, the Total Hazard Quotient Index (THQI) of non-carcinogenic risk was evaluated by adding the values of HQ_nitrate_ and HQ_flouride_:

$$\mathrm{THQI }= {\mathrm{HQ}}_{\mathrm{nitrate}} + {\mathrm{HQ}}_{\mathrm{fluoride}}$$

The samples with HQ and THQI above 1 may cause non-carcinogenic risk and they are considered inappropriate for ingestion. Samples with HQ and THQI below 1 are considered safe for consumption (e.g., Narsimha and Rajitha 2018; He et al. 2019).

## Results and discussion

### Hydrochemistry

Lake water (*n* = 3): They are slightly acidic to almost neutral with pH varying between 5.9 and 7.3 (average: 6.7; Table 2). EC changes between 294 and 536 μS/cm (average: 454 μS/cm) and TDS fluctuates between 147 and 269 mg/L (average: 227 mg/L). All the samples have TDS < 1000 mg/L, and they are categorized as fresh (e.g., Freeze and Cherry 1979). Among the cations, Na is more abundant (52–53.8 mg/L; average: 52.9 mg/L) than the almost comparable K (22.8–28.5 mg/L; average: 25.2 mg/L), Ca (21.2–26.1 mg/L; average: 22.9 mg/L), and Mg (21.3–21.9 mg/L; average: 21.7 mg/L). Among the major anions, the concentration of HCO_3_ (256.8–310.2 mg/L; average: 276.5 mg/L) is more compared to SO_4_ (36.3–39.2 mg/L; average: 38.2 mg/L), and Cl shows an abundance of 14.2–18 mg/L (average: 15.6 mg/L). NO_3_ remains below the detection limit (< 0.75 mg/L) in all the samples and F fluctuates between 0.3 and 4.3 mg/L (average: 1.8 mg/L; Table 2).

**Table 2:** Physicochemical parameters of lake and groundwater from and around Lake Coatetelco (central-south Mexico) with respect to WHO (2017)-recommended values. Number (%) of samples above the permissible limits is shown (*LD*, limit of detection)

	Lake water (*n* = 3)			Groundwater (*n* = 17)			WHO (2017)
	Min–Max	Avg	Sample > WHO (2017) limits Number (%)	Min–Max	Avg	sample > WHO (2017) limits Number (%)	
pH	5.9–7.3	6.7	1 (33%)	5.8–7.3	6.8	0 (0%)	6.5–8.5
EC (µS/cm)	294–536	453.7	0 (0%)	481–2030	926.6	1 (6%)	1500
TDS (mg/L)	147–269	226.7	0 (0%)	242–1020	463.6	1 (6%)	1000
Ca^2+^ (mg/L)	21.2–26.1	22.9	0 (0%)	34.3–75	53.3	0 (0%)	75
Mg^2+^ (mg/L)	21.3–21.9	21.7	0 (0%)	12–64.2	28.3	2 (12%)	50
Na^+^ (mg/L)	52–53.8	52.9	0 (0%)	20.5–308.7	103.6	2 (12%)	200
K^+^ (mg/L)	22.8–28.5	25.2	3 (100%)	3.6–46.8	13.5	5 (29%)	12
HCO_3_^−^ (mg/L)	256.8–310.2	276.5	1 (33%)	285.4–1102.2	559.2	12 (82%)	300
Cl^−^ (mg/L)	14.2–18	15.6	0 (0%)	4.3–90.3	27.9	0 (0%)	250
SO_4_^2−^ (mg/L)	36.3–39.2	38.2	0 (0%)	18.7–198.6	69.5	0 (0%)	250
NO_3_^−^ (mg/L)	< LD	-	-	< LD–29.5	8.7	0 (0%)	50
F^−^ (mg/L)	0.3–4.3	1.8	1 (33%)	0.1–8.7	1.9	9 (53%)	1.5

Groundwater (*n* = 17): Some samples (*n* = 5) are slightly acidic, and the rest are almost neutral with pH between 5.8 and 7.3 (average: 6.8). The acidic nature of some samples may be due to abundant volcanic deposits constituting the aquifer and degradation of organic carbon in the soil layers. EC remains heterogeneous and higher compared to the lake water (481–2030 μS/cm; average: 926.6 μS/cm). TDS of 242–1020 mg/L (average: 463.6 mg/L) classifies one groundwater sample as slightly brackish (TDS > 1000 mg/L) and the rest as fresh (TDS < 1000 mg/L; Freeze and Cherry 1979). Among the cations, Na is more abundant (20.5–308.7 mg/L; average: 103.6 mg/L) than Ca (34.3–75 mg/L; average: 53.3 mg/L), Mg (12–64.2 mg/L; average: 28.3 mg/L), and K (3.6–46.8 mg/L; average: 13.5 mg/L). Among the major anions, the concentration of HCO_3_ (285.4–1102.2 mg/L; average: 559.2 mg/L) is more compared to SO_4_ (18.7–198.6 mg/L; average: 69.5 mg/L) and Cl (4.3–90.3 mg/L; average: 27.9 mg/L). NO_3_ remains heterogenous with below the detection limit (< 0.75 mg/L) in some samples (*n* = 6) and up to 29.5 mg/L (average: 8.7 mg/L) in others (*n* = 11). F fluctuates between 0.1 and 8.7 mg/L with an average of 1.9 mg/L.

### Rock-water interaction

**Fig. 2:**
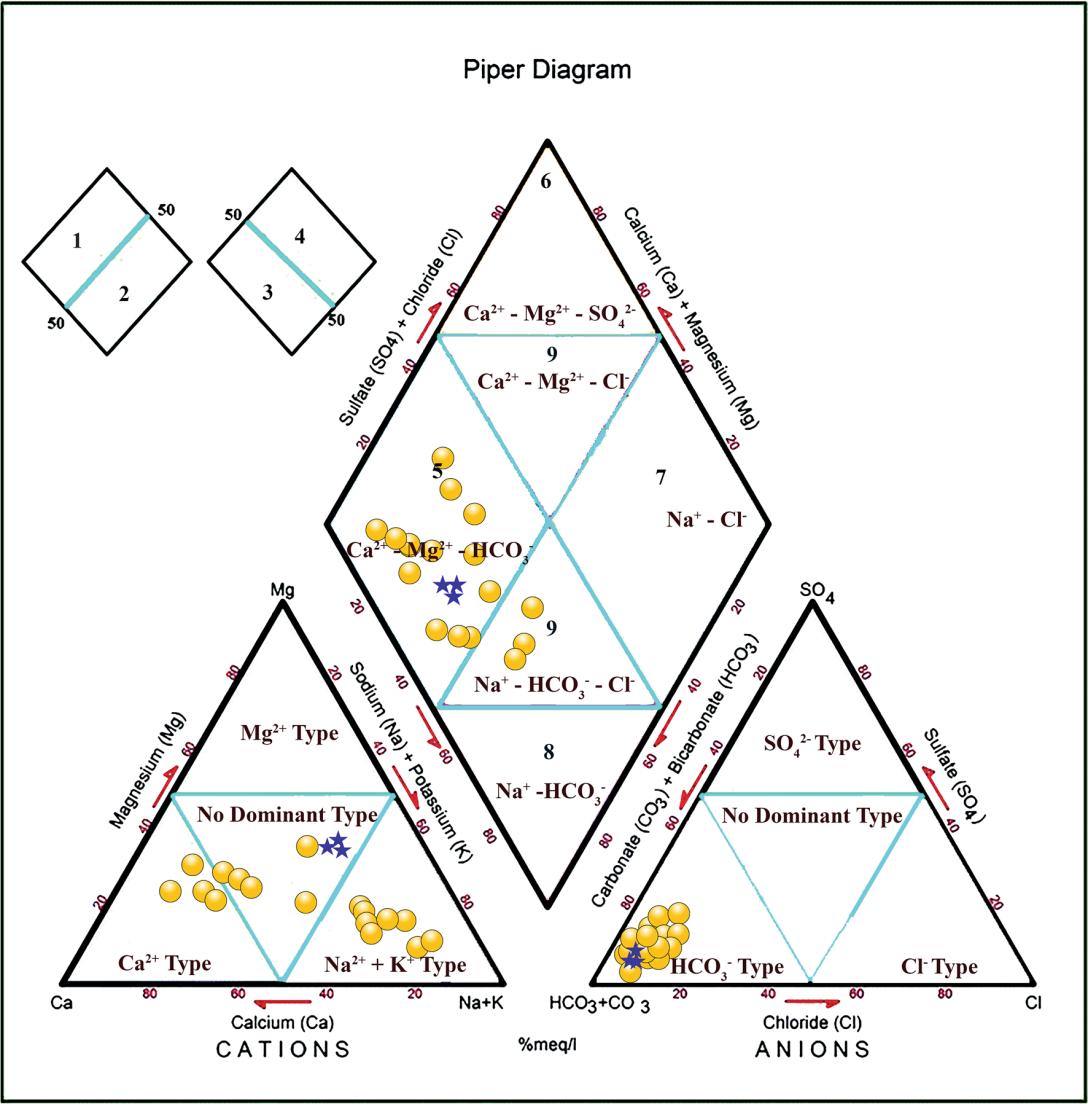
Classification of hydrochemical facies of the lake (blue star) and groundwater (yellow circle) from and around Lake Coatetelco (central-south Mexico) evaluated in the trilinear Piper diagram

**Fig. 3:**
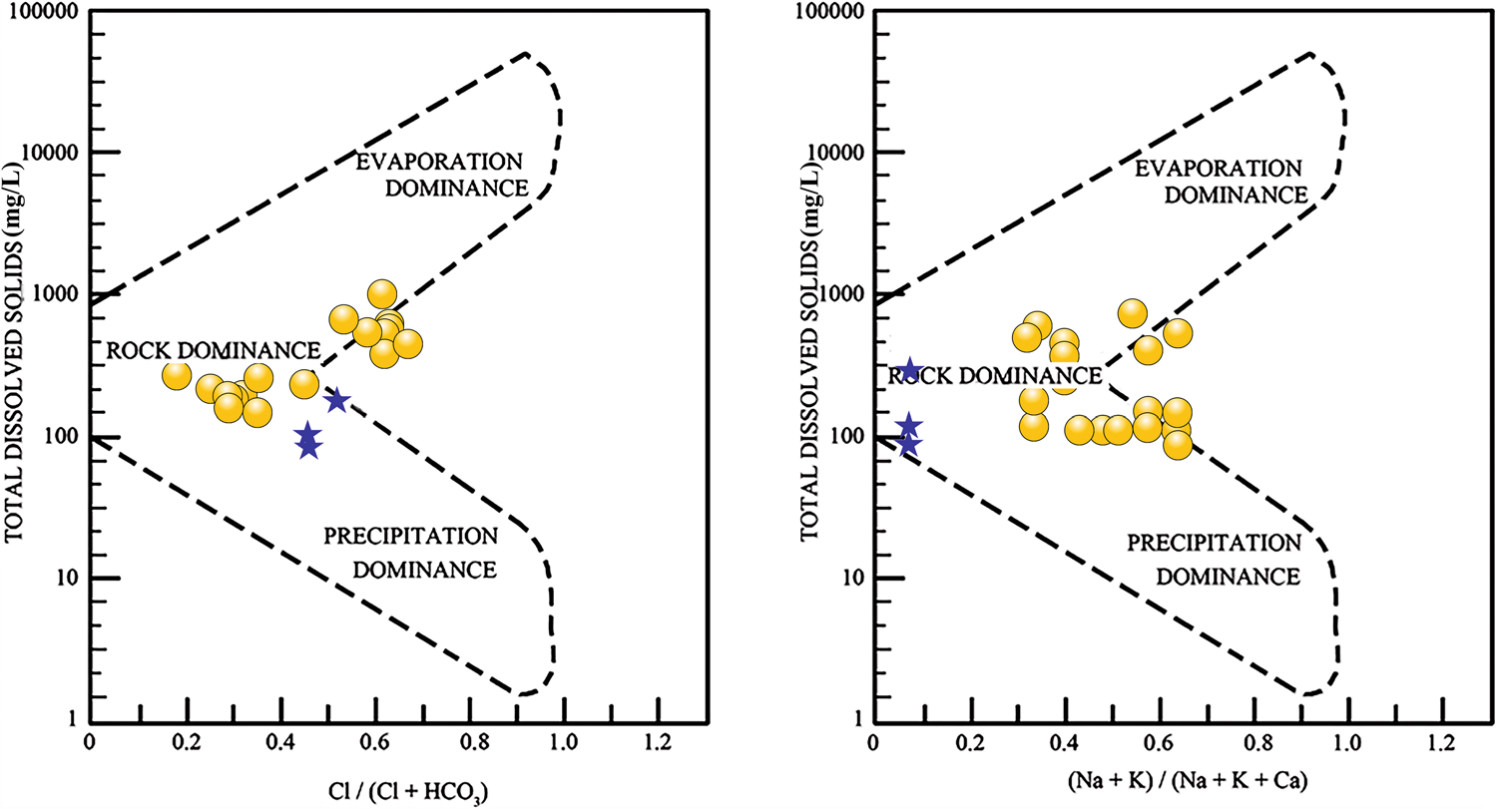
Evaluation of the influences of precipitation, evaporation, and rock-water interaction on lake (blue star) and groundwater (yellow circle) samples from and around the Lake Coatetelco (central-south Mexico) in Gibbs diagrams

Lake water (*n* = 3): Cation (Ca, Mg, and Na + K) and anion (HCO_3_, SO_4_, and Cl) abundances in the trilinear diagram of Piper (1944) grouped the samples in Ca-Mg-HCO_3_ water type (Fig. 2). The distributions of cation classified the lake water as no dominant type, indicating interactions with variable watershed geology, and the anions grouped the samples as dominant HCO_3_ type due to the importance of carbonic acid (H_2_CO_3_) in weathering reactions (Fig. 2). The major influence of rock-water interaction compared to precipitation and evaporation on the hydrochemistry of lake water was mirrored by comparing the ratio of Na + K/(Na + K + Ca) against TDS and the ratio of Cl/(Cl + HCO_3_) against TDS in Gibbs (1970) diagrams (Fig. 3). Both the Piper and Gibbs plots suggest that the interaction with catchment lithologies controlled the lake water chemistry. Abundant HCO_3_ in the lake water reflected the interaction with watershed lithologies such as limestone and volcanic deposits (basalt and andesite) as well as non-carbonate rocks such as the alluvial deposits (e.g., Fries 1960; Rivera-Carranza et al. 1998; CONAGUA 2020). Additionally, the oxidation of organic matter present in the topsoil might have also contributed HCO_3_ through the CO_2_ dissolution in water. The weathering of volcanic deposits such as basalt and andesite produces water dominated by HCO_3_, Ca, and Mg, similar to Ca-Mg-HCO_3_ type of water from Lake Coatetelco (Eugster and Hardie 1978).

Groundwater (*n* = 17): The diamond grid in Piper trilinear diagram (Fig. 2) reflects heterogeneity of ion concentrations and classifies some groundwater samples as Na-HCO_3_-Cl water type (*n* = 3) and the rest as Ca-Mg-HCO_3_ (*n* = 14). All the samples are of dominant HCO_3_ type in the anion triangle and fluctuate between Ca and Na + K water types in the cation triangle (Fig. 2). The samples with dominant bicarbonate and Ca mirror the interactions with limestone in deeper part of the aquifer, and the samples with no dominant cation as well as dominant Na + K reflect the intercalations with sedimentary (sandstone and conglomerate) and volcanic deposits constituting the upper part of aquifer lithology (e.g., Fries 1960; Rivera-Carranza et al. 1998; CONAGUA 2020). The Gibbs diagrams (Fig. 3) reflect the interaction of groundwater with catchment lithologies with lower Na + K/(Na + K + Ca) values indicating interaction with limestone and higher Na + K/(Na + K + Ca) values representing more interaction with the silicate-bearing (volcanic) deposits (e.g., Marandi and Shand 2018). In the sample with higher Cl/Cl + HCO_3_ (*n* = 8), the irrigation return flow possibly enriched Cl and the Piper diagram grouped some of them in the Na-HCO_3_-Cl facies (e.g., Nagarajan et al. 2010). These wells with Cl between 57.1 and 90.3 mg/L are present at the northern lake margin with more agricultural activity.

### Drinking water quality

#### Comparison with WHO guideline

Lake water (*n* = 3): Physical parameters remain within the recommendations of WHO (2017), except for the pH of one sample (Table 2). The sample with pH of 5.9 is more acidic compared to the suggested range (pH: 6.5–8.5). With respect to the major ions, all samples (100%) have K above the permissible limit for drinking (> 12 mg/L) and HCO_3_ of only one sample (33%) exceeds the allowable content (> 300 mg/L). The basic sources of potassium are silicate minerals, i.e., K-feldspars, muscovite, and clay mineral (kaolinite) in the alluvium as well as abundant volcanic deposits in watershed, which are dissolved by the low pH (carbonic acid) water flowing into the lake. The acidic nature of inflowing water is mirrored by pH < 7 in two out of the three samples. More bicarbonate than the permissible limit for drinking in one sample could be from the interaction of inflowing water with organic-rich soil layers and additionally from higher dissolution of limestone in the watershed (e.g., Venkatramanan et al. 2017; Chung et al. 2020).

Concentrations of Ca (< 75 mg/L), Mg (< 50 mg/L), Na (< 200 mg/L), Cl (< 250 mg/L), and SO_4_ (< 250 mg/L) of all samples remain within the permissible limits (Table 2). NO_3_ concentrations remain below the permissible limit of WHO (50 mg/L) and Mexican norms (42 mg/L) in all the samples. About 33% samples have fluoride above the recommended limit of 1.5 mg/L and the remaining 66% samples contain fluoride within the WHO (2017) guidelines. The samples show possibility to generate dental caries (< 0.5 mg/L, 33%), beneficial for human health (0.6–1.5 mg/L, 33%), and vulnerability of dental and skeletal fluorosis (> 2 mg/L, 33%) (e.g., Adimalla 2020).

Groundwater (*n* = 17): All the samples have pH within the permissible limit for drinking. Only one sample (6%) with TDS of 1020 mg/L and EC of 2030 μS/cm has physical characteristics above the WHO-recommended values (Table 2). About 82% samples (*n* = 12) have HCO_3_ above the permissible limit (300 mg/L). Similarly, 29% (*n* = 5) samples have K (12 mg/L) and 12% (*n* = 2) samples have Mg (50 mg/L) and Na (200 mg/L) exceeding the permissible values. More bicarbonate than the permissible limit in most of the samples shows the effect of limestone dissolution (e.g., Venkatramanan et al. 2017; Chung et al. 2020). The sources of higher potassium and magnesium in some samples are chemical weathering of both the felsic and mafic silicate minerals like K-feldspar, and amphibole present in the volcanic deposits by the low pH groundwater.

Concentrations of Ca (< 75 mg/L), Cl (< 250 mg/L), and SO_4_ (< 250 mg/L) of all the samples are within the permissible limits (Table 2). NO_3_ concentrations remain below the permissible limit of WHO (50 mg/L) as well as the Mexican norms (42 mg/L). About 53% samples (*n* = 9) have fluoride above the recommended limit of 1.5 mg/L and 47% samples (*n* = 8) might be responsible for dental and skeletal fluorosis (> 2 mg/L; Adimalla 2020). The other samples can be grouped as possible trigger to dental caries (< 0.5 mg/L, 47%; Adimalla 2020). Higher fluoride in more than half of the groundwater samples reflects possible weathering of fluoride-bearing minerals present in the limestone and volcanic deposits through the rock-water interactions. Carrillo-Rivera et al. (2002) also reported up to 3.7 mg/L of F in the groundwater of San Luis Potosi state and Reyes-Gómez et al. (2017) observed up to 6 mg/L of fluoride in the groundwater of Chihuahua state. Both the regions with volcanic geology showed F in groundwater comparable to the groundwater in surroundings of the Lake Coatetelco (up to 8.7 mg/L). Several wells of previous studies in central and northern Mexico as well as the present evaluation in central-south Mexico showed F exceeding the WHO-recommended value of 1.5 mg/L.

#### Drinking water quality index

The groundwater extracted through shallow borewells as well as the lake water are presently used for drinking as well as other domestic usages. The drinking water quality index (DWQI) grouped them into five categories such as excellent (< 50), good (50–100), poor (100–200), very poor (200–300), and unsuitable (> 300) (Table 3). In general, most of the lake water as well as groundwater samples are in excellent and good categories.

**Table 3:** General classifications of the water samples (number and %) from and around Lake Coatetelco (central-south Mexico) based on the drinking water quality index (DWQI)

DWQI	Categories	Lake water (*n* = 3)	Groundwater (*n* = 17)
		No. of samples (%)	No. of samples (%)
< 50	Excellent	3 (100%)	8 (47%)
50–100	Good	-	8 (47%)
100–200	Poor	-	1 (6%)
200–300	Very poor	-	-
> 300	Unsuitable	-	-

Lake water (*n* = 3): About 67% (*n* = 2) samples (DWQI: 36.6–37.3) are grouped in excellent category and one sample (33%) with DWQI of 68.1 is in the good category even though K in all the samples and F in one sample remain above the WHO permissible limits (Table 3).

Groundwater (*n* = 17): DWQI values (28.5–159.9) are heterogenous compared to the lake water. About 41% samples (*n* = 7) are in excellent category and 53% (*n* = 9) are in good category (Table 3). Only one sample (6%) is in poor category, and it has the highest values of F (8.7 mg/L), TDS (1020 mg/L), and Na (308.7 mg/L) among all the lake and groundwater samples.

### Irrigation water quality

#### Indices

**Fig. 4:**
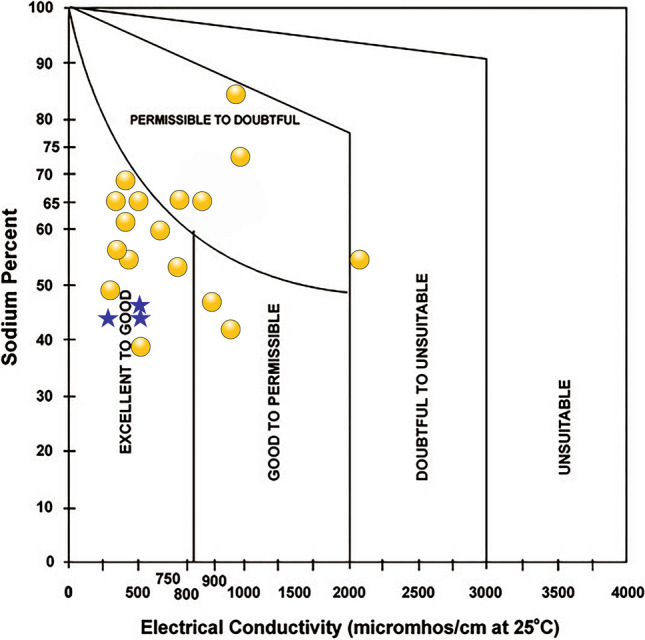
Classification of lake (blue star) and groundwater (yellow circle) from and around the Lake Coatetelco (central-south Mexico) for irrigation suitability in the salinity hazard vs. alkali hazard index of the USSL diagram

**Fig. 5:**
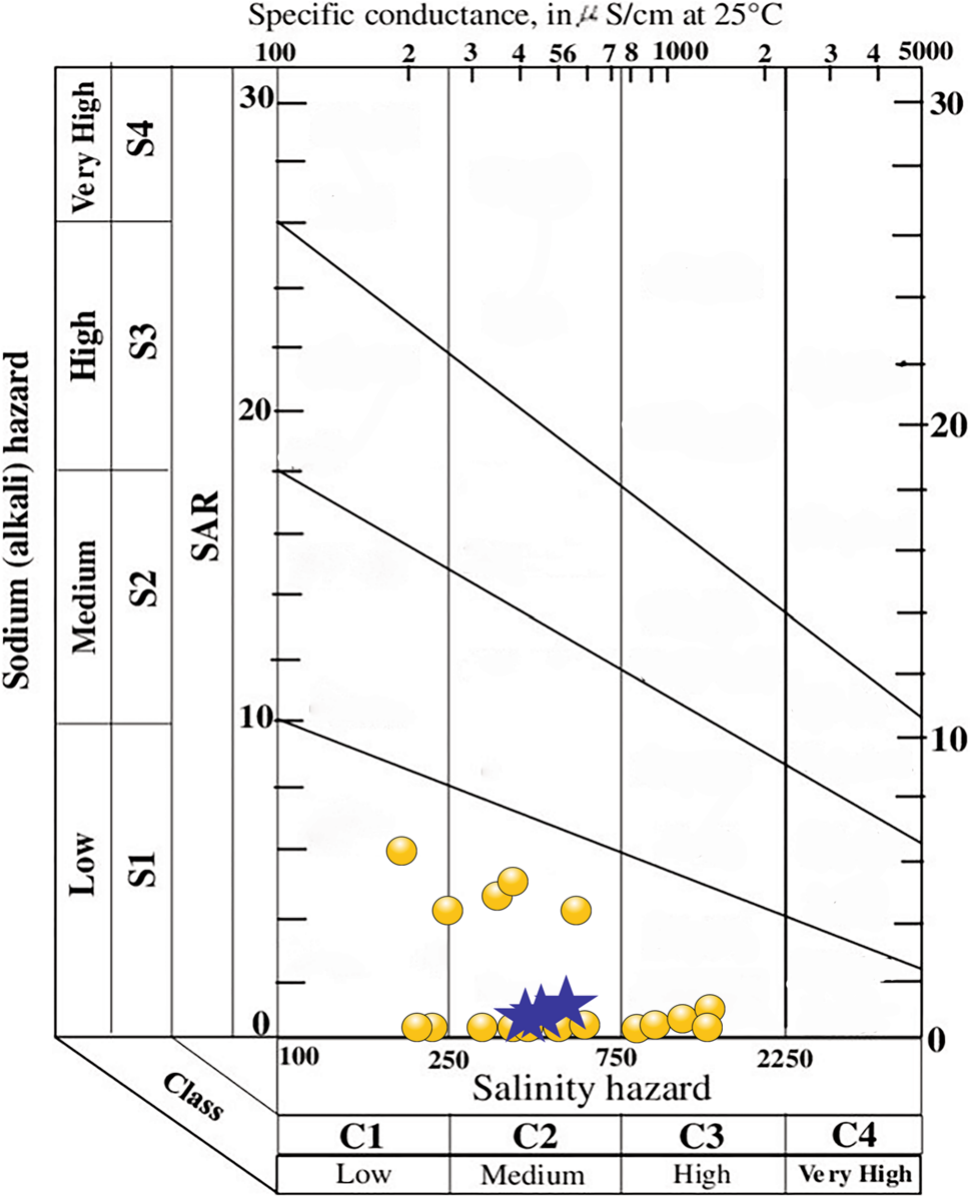
Irrigation suitability of lake and groundwater samples from and around the Lake Coatetelco (central-south Mexico) in Wilcox diagram

**Fig. 6:**
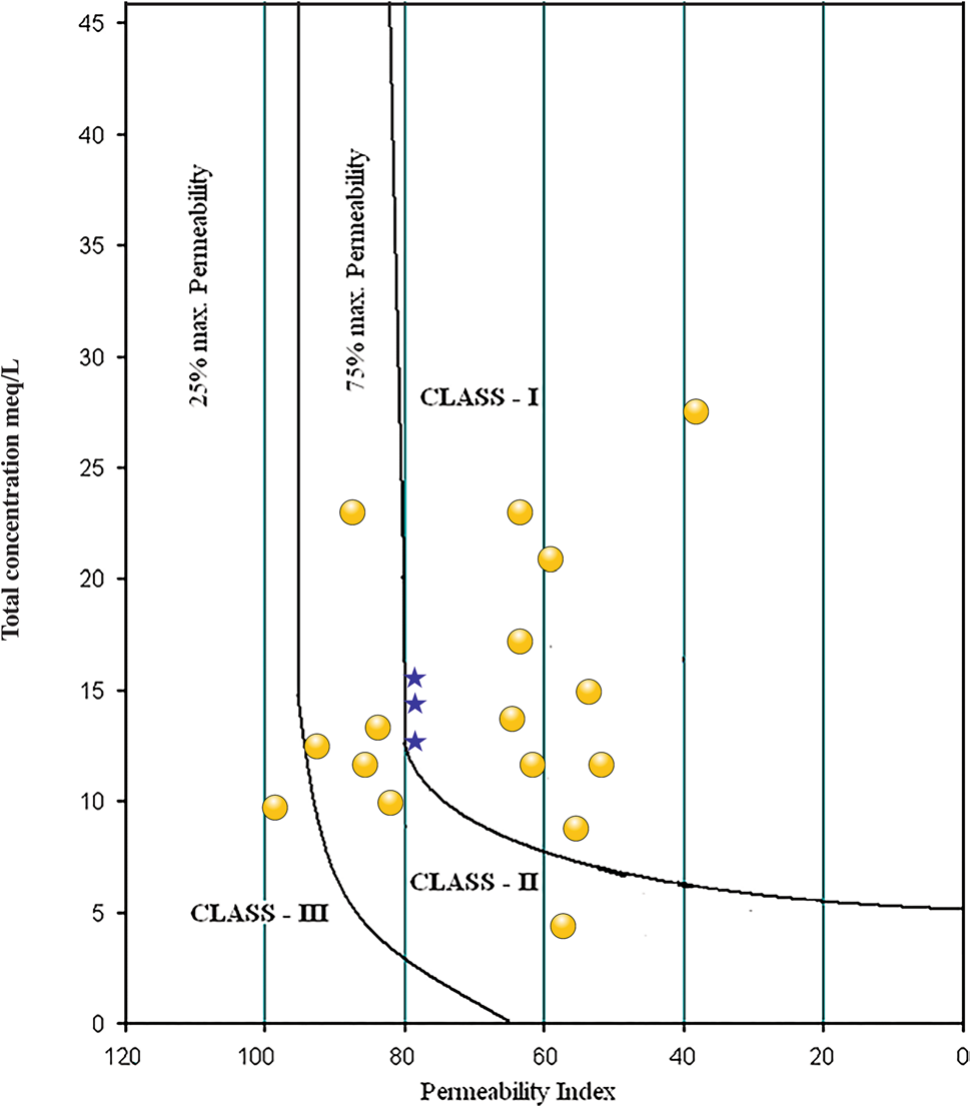
Classification of lake (blue star) and groundwater (yellow circle) samples from and around the Lake Coatetelco (central-south Mexico) based on the permeability index values in the Doneen classification

The lake water is moderately hard (67%, TH: 75–150) and hard (33%, TH: 150–300) (e.g., Sawyer and McCarty 1967). Among the groundwater samples, about 77% are hard, 17% are very hard (TH > 300), and 6% are moderately hard (Table 4). The estimation of salinity and alkalinity hazards through indices such as RSC, SAR, Na%, KR, MR, PS, and PI evaluated the suitability of both lake water and groundwater for irrigation (e.g., Eaton 1950; Richards 1954; Doneen 1962, 1964; Kelley 1963; Sawyer and McCarty 1967; Paliwal 1972). RSC represents the amount of carbonate (NaCO_3_ and NaHCO_3_) present in the irrigation water and its higher values (RSC > 2.5) reflect an increase in sodium adsorption. Irrigation with more saline water alters the osmotic pressure and affects the crop yield (e.g., Thorne and Peterson 1954; Trivedy and Goel 1984). It increases the kinematic viscosity and prevents water from reaching the branches and leaves of plants by decreasing the permeability coefficient. High salinity water also induces the accumulation of salt in soil, and the high sodium concentration leads to enhancement in soil alkalinity. It reduces the stability of soil structure and affects the extension of plant roots and movement of water through the soil. SAR estimated the potential of Na accumulation in soil through the Na-enriched irrigation water with low SAR (< 10) values reflecting the better water percolation time (Richards 1954). In the US Regional Salinity Laboratory (USSL) diagram, SAR values grouped all the lake and groundwater as low sodium hazard (S1), suggesting their suitability for all types of soils and crops (Fig. 4). EC (Wilcox 1955), however, divided the samples between low salinity hazard (C1) and high salinity hazard (C3). The samples in low salinity hazard are suitable for irrigating all plants, the samples in medium salinity hazard are suitable for salt-tolerant plants, and the samples with high salinity hazards are unsuitable for irrigation (Table 4). The Wilcox diagram (Na% vs. EC) classified the samples in categories varying from excellent to doubtful for irrigation (Fig. 5, e.g., Wilcox 1955). Similarly, the permeability index (PI) vs. total concentration (meq/L) in the Doneen (1964) classification divided the samples between class I with > 75% of permeability and class III with < 25% of permeability to assess the irrigation suitability (Fig. 6).

**Table 4:** Classification of water samples (number and %) from and around Lake Coatetelco (central-south Mexico) on different irrigation indices

Index	Range	Suitability class	Lake water (*n* = 3)	Groundwater (*n* = 17)
			Number of samples (%)	Number of samples (%)
TH (Sawyer and McCarty 1967)	< 75	Soft	-	-
	75–150	Moderately high	2 (67%)	1 (6%)
	150–300	Hard	1 (33%)	13 (77%)
	> 300	Very hard	-	3 (17%)
Salinity hazard (EC μS/cm)	< 250	Excellent	-	-
	250–750	Good	3 (100%)	8 (47%)
	750–2250	Permissible	-	9 (53%)
	2250–3000	Doubtful	-	-
	> 3000	Unsuitable	-	-
SAR (Richards 1954)	< 10	excellent	3 (100%)	17 (100%)
	10–18	good	-	-
	18–26	doubtful	-	-
	> 26	Unsuitable	-	-
RSC (Eaton 1950)	< 1.25	Excellent	-	1 (6%)
	1.25–2.50	Acceptable	3 (100%)	7 (41%)
	> 2.50	Unsuitable	-	9 (53%)
Na% (Wilcox 1955)	< 20	Excellent	-	2 (12%)
	20–40	Good	-	6 (35%)
	40–60	Permissible	3 (100%)	5 (29%)
	60–80	Doubtful	-	4 (24%)
	> 80	Unsuitable	-	-
KR (Kelley 1963)	< 1	Suitable	3 (100%)	10 (59%)
	> 1	Unsuitable	-	7 (41%)
MR (Paliwal 1972)	< 50	Acceptable	-	10 (59%)
	> 50	Not acceptable	3 (100%)	7 (41%)
PS (Rawat et al. 2018)	< 3	Safe	3 (100%)	15 (88%)
	> 3	Unsafe	-	2 (12%)
PI (Doneen 1964)	> 75	Good	3 (100%)	8 (47%)
	25–75	Fair	-	9 (53%)
	< 25	Poor	-	-

Lake water (*n* = 3): Based on the salinity hazard (EC: 250–750 in μS/cm, good) and SAR (< 10, excellent), all the lake water samples are suitable for irrigation. RSC (1.25–2.50) and Na% (40–60) ranges also group them in acceptable and permissible categories (Table 4). The indices of Kelly ratio (KR), potential salinity (PS), and permeability (PI) categorized all as suitable, safe, and good for irrigation. These samples in C2S1 field of USSL suggest moderate salinity hazard and low sodium hazard (C2S1), and all of them can be used to irrigate crops without considering any special measure (Fig. 4). They are also excellent-to-good for irrigation in the Wilcox diagram (Fig. 5). The Doneen diagram classified all these samples under class I with > 75% of permeability, indicating their suitability for irrigation (Fig. 6).

Groundwater (*n* = 17): The individual indices as well as classification diagrams show the heterogeneity of groundwater. The salinity hazard divides the samples in good (EC: 250–750 in μS/cm, 47%) and permissible (EC: 750–2250 μS/cm, 53%) categories. SAR (< 10) classifies all of them (100%) as excellent for irrigation (Table 4). Based on RSC (> 2.50), about 53% of samples (*n* = 9), however, are unsuitable. Similarly, about 24% (*n* = 4) are doubtful in Na% (60–80) index (Table 4). The Kelly ratio (KR < 1) classifies 59% (*n* = 10) samples as suitable, the potential salinity (PS < 3) groups 88% (*n* = 15) samples as safe, and the permeability index (PI) groups 47% (*n* = 8) as good and 53% (*n* = 9) as fair for irrigation. In the USSL classification, about 71% samples (*n* = 12) with low and medium salinity hazards (C1S1 and C2S1) can be used to irrigate crops without considering any special measure, and 29% samples (*n* = 5) with high salinity hazard (C3S1) are generally suitable for salt-tolerant plants (Fig. 4). Irrigation with the high salinity hazard water might affect crop growth through osmotic effects and nutritional disorders (e.g., Läuchli and Epstein 1990). Irrigation with the water of this nature needs special consideration for salinity control and mechanism to improve the soil permeability and should be considered only to irrigate salt-tolerant crops under favorable drainage conditions. The Wilcox diagram also classifies 71% of groundwater samples (*n* = 12) as excellent-to-good (*n* = 10) and good-to-permissible (*n* = 2). The remaining 29% samples are in the permissible-to-doubtful (*n* = 4) and doubtful-to-unsuitable (*n* = 1) categories for irrigation (Fig. 5). They are also in the excellent-to-good groups for irrigation in the Wilcox diagram. The Doneen diagram classifies 59% samples (*n* = 11) under class I with > 75% of permeability and 35% (*n* = 6) in class II with 25–75% permeability, indicating their suitability for irrigation (Fig. 6). One sample, in class 3 with < 25% permeability, is unsuitable for irrigation.

#### Irrigation water quality index (IWQI)

Estimation of IWQI assessed the overall quality for irrigation, and demarcation of the most suitable wells. It divides the groundwater samples into five different classes such as severe restriction (< 40), high restriction (40–55), moderate restriction (55–70), low restriction (70–85), and no restriction (85–100) (Table 5).

**Table 5:** Irrigation suitability of water samples (number and %) from and around Lake Coatetelco (central-south Mexico) based on the irrigation water quality index (IWQI) classification

IWQI	Categories	Restriction categories	Lake water (*n* = 3)	Groundwater (*n* = 17)
			No. of samples (%)	No. of samples (%)
85–100	I	No restriction	-	-
70–85	II	Low restriction	-	-
55–70	III	Moderate restriction	3 (100%)	8 (47%)
40–55	IV	High restriction	-	8 (47%)
0–40	V	Severe restriction	-	1 (6%)

Lake water (*n* = 3): All have almost similar and homogenous IWQI between 64.3 and 64.4 (average: 78) and they are in the group of moderate restriction for irrigation. There are no sample in the high to severe restriction categories (Table 5). The lake water should be used for irrigating soils with moderate to high permeability with special salinity control practices to minimize salt accumulation.

Groundwater (*n* = 17): The samples with variable IWQI (38.8–68.6; average: 54.5) are in the categories of moderate restriction (47%, *n* = 8), high restriction (47%, *n* = 8), and severe restriction (6%, *n* = 1). The groundwater samples are relatively less suitable for irrigation compared to the lake water. It could be used for irrigating soils with light texture or moderate permeability to avoid the effects of salt leaching. The salt sensitivity plants should be avoided in this region and the soil texture can be modified by changing the sand to clay ratio.

### Health risk assessment

**Fig. 7:**
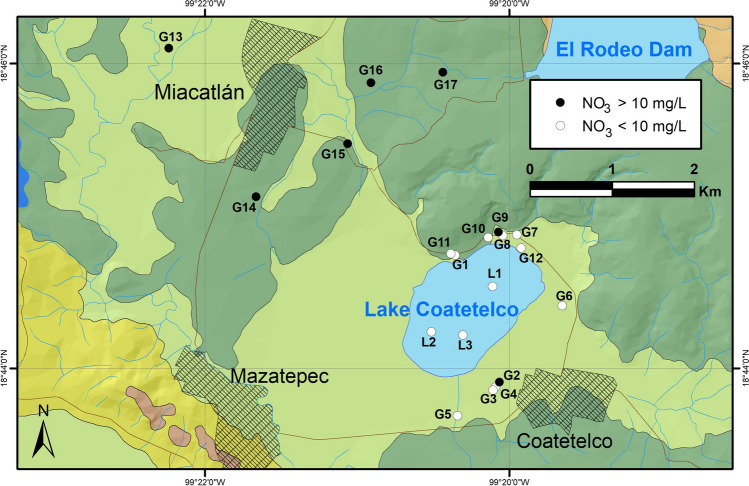
Distribution of the lake water samples and groundwater wells in surroundings of the Lake Coatetelco (central-south Mexico) with NO_3_ < 10 mg/L and NO_3_ > 10 mg/L

The exposure through ingestion of lake water and groundwater with elevated concentrations of NO_3_ could cause methemoglobinemia (commonly known as blue baby syndrome in infants; Adimalla et al. 2018; Karunanidhi et al. 2020b) and higher F might trigger both dental and skeletal fluorosis (e.g., Kimambo et al. 2019; Alarcón-Herrera et al. 2020). We computed non-carcinogenic risks for the population of different age groups residing around the Lake Coatetelco from ingestion of nitrate and fluoride in water by considering an adult male with an average weight of 74 kg, adult female with an average weight of 68 kg, and a child with average weight of 20 kg in Table 6. The estimated Health Quotients (HQ_nitrate_ and HQ_fluoride_) for individuals suggest absence of any risk from NO_3_ in both lake and groundwater. However, the HQ_fluoride_ shows potential non-carcinogenic concerns and possible effect in order of child > adult female > adult male. In the lake water samples, the HQ_nirate_ values for different age groups could not be computed as nitrate remained below the detection limit. However, the HQ_fluoride_ values for adult male (0.19–3.11; avg: 1.29), adult female (0.21–3.39; avg: 1.40), and child (0.22–3.59, avg: 1.48) suggest relatively higher but similar risks for child and adult female compared to the adult male in 33% of the samples (HQ > 1). In the groundwater samples, the HQ_nitrate_ outcome for adult male (0.04–0.81; avg: 0.37), adult female (0.05–0.88; avg: 0.40), and child (0.04–0.74; avg: 0.34) remained < 1 and did not show any immediate health risk. Negative correlation (*r* =  − 0.62) between NO_3_ and Cl in groundwater and nitrate remaining below the detection limit (< 0.75 mg/L) in most of the samples with Cl above 20 mg/L rules out the domestic sewage and farm manures as the dominant sources of nitrate (e.g., Mahlknecht et al. 2008). The synthetic nitrogenous fertilizers (e.g., ammonium nitrate) used in the agricultural fields near the groundwater wells and lake surroundings possibly contributed nitrate. The groundwater samples with NO_3_ < 10 mg/L generally show natural conditions, with minimal human interferences around the lake, and the samples with > 10 mg/L of nitrate (*n* = 7) around the Miacatlán village indicate anthropogenic impact possibly from synthetic fertilizers on a part of the shallow aquifer (Fig. 7, e.g., Marghade et al. 2015; Subba Rao et al. 2020). Unlike the groundwater of Monterrey City, the effects of sewage leakage and soil organic nitrogen were minimal on groundwater around the Lake Coatetelco (e.g., Torres-Martinez et al. 2020). All the samples of this study, however, remained below the WHO (< 50 mg/L) norms and did not pose any immediate health risk to the surrounding population (e.g., Adimalla et al. 2018).

**Table 6:** Computed value of HQ for nitrate and fluoride in water from and around Lake Coatetelco (central-south Mexico) and THQI values for male, female, and child with average weights of 74 kg, 68 kg, and 20 kg, respectively

Sample	HQ_nitrate_			HQ_fluoride_			THQI		
	Male	Female	Child	Male	Female	Child	Male	Female	Child
Lake water									
1	-	-	-	0.56	0.61	0.64	0.56	0.61	0.64
2	-	-	-	**3.11**	**3.39**	**3.59**	**3.11**	**3.39**	**3.59**
3	-	-	-	0.19	0.21	0.22	0.19	0.21	0.22
Minimum	-	-	-	0.19	0.21	0.22	0.19	0.21	0.22
Maximum	-	-	-	3.11	3.39	3.59	3.11	3.39	3.59
Average	-	-	-	1.29	1.40	1.48	1.29	1.40	1.48
Groundwater									
1	0.24	0.26	0.22	0.12	0.13	0.14	0.35	0.39	0.35
2	0.35	0.38	0.32	**1.95**	**2.12**	**2.24**	**2.29**	**2.49**	**2.56**
3	0.25	0.27	0.23	**1.67**	**1.81**	**1.92**	**1.92**	**2.09**	**2.15**
4	0.25	0.27	0.23	**2.58**	**2.81**	**2.98**	**2.84**	**3.09**	**3.21**
5	0.04	0.05	0.04	0.34	0.37	0.39	0.38	0.42	0.43
6	-	-	-	0.29	0.32	0.34	0.29	0.32	0.34
7	-	-	-	0.23	0.25	0.26	0.23	0.25	0.26
8	-	-	-	**6.35**	**6.91**	**7.32**	**6.35**	**6.91**	**7.32**
9	0.34	0.37	0.31	0.28	0.30	0.32	0.61	0.67	0.63
10	-	-	-	0.29	0.32	0.34	0.29	0.32	0.34
11	-	-	-	**2.15**	**2.34**	**2.47**	**2.15**	**2.34**	**2.47**
12	-	-	-	**2.30**	**2.50**	**2.65**	**2.30**	**2.50**	**2.65**
13	0.35	0.39	0.33	**1.12**	**1.22**	**1.29**	**1.47**	**1.60**	**1.62**
14	0.54	0.59	0.50	**1.55**	**1.69**	**1.79**	**2.09**	**2.27**	**2.29**
15	0.81	0.88	0.74	0.09	0.10	0.11	0.90	0.98	0.85
16	0.47	0.51	0.43	**1.83**	**1.99**	**2.11**	**2.30**	**2.50**	**2.54**
17	0.39	0.42	0.36	0.11	0.12	0.13	0.50	0.54	0.49
Minimum	0.04	0.05	0.04	0.09	0.10	0.11	0.23	0.25	0.26
Maximum	0.81	0.88	0.74	6.35	6.91	7.32	6.35	6.91	7.32
Average	0.37	0.40	0.34	1.37	1.49	1.57	1.60	1.74	1.79

Bold values indicate HQ > 1

**Fig. 8:**
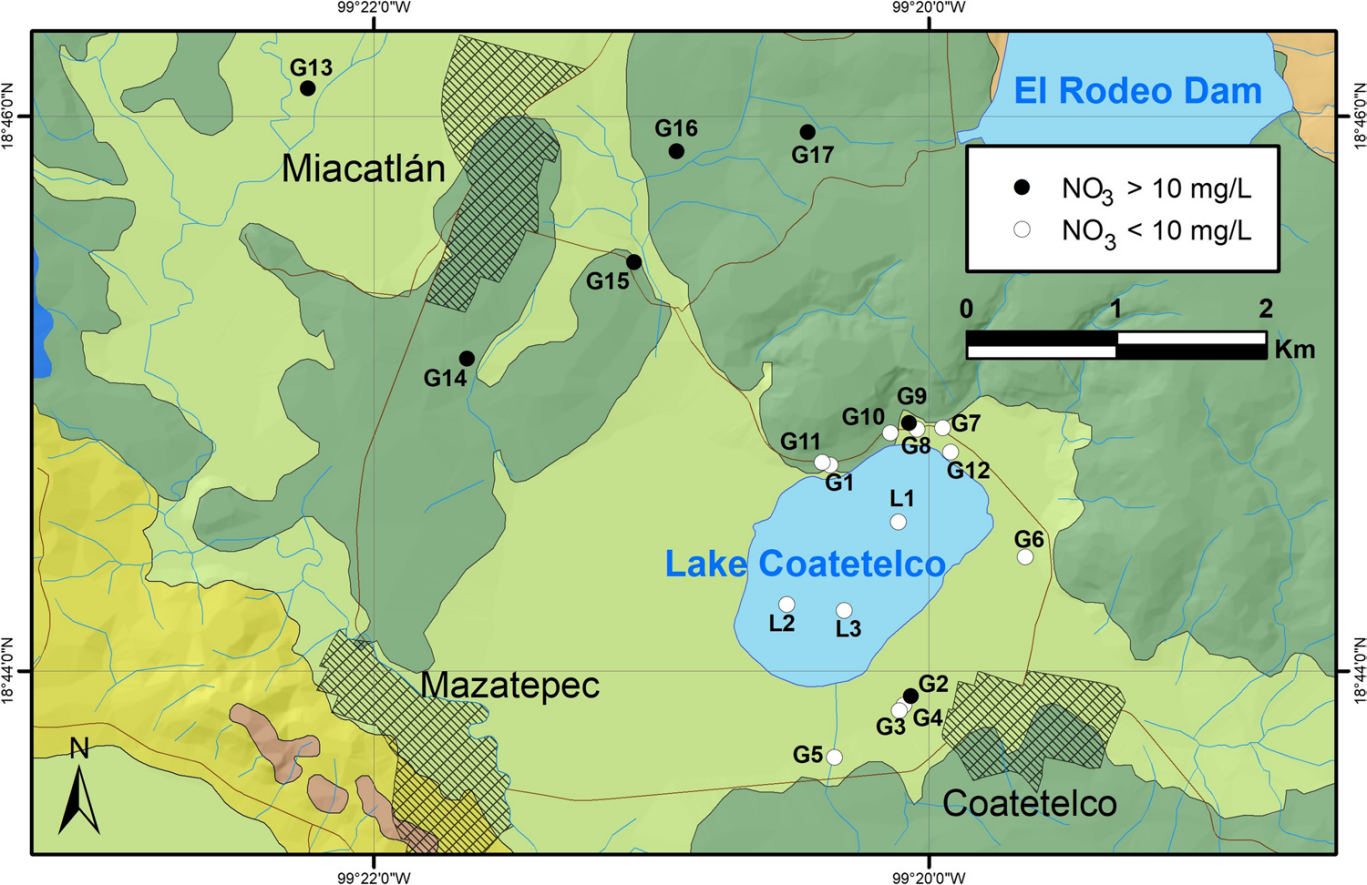
Distribution of the lake water samples and groundwater wells in surroundings of the Lake Coatetelco (central-south Mexico) with HQ_fluoride_ > 1 (*n* = 9, 53%) with possible non-carcinogenic risk for adult and child population of this region

HQ_fluoride_ values, however, showed possible risk of dental and skeletal fluorosis from consumption of 53% of the groundwater samples for male (0.09–6.35; avg: 1.37), female (0.10–6.91; avg: 1.49), and child (0.11–7.32; avg: 1.57). In the present study, HQ_fluoride_ for non-carcinogenic risk exceeds 1 in 33% of lake water samples and 53% of groundwater samples with respect to men, women, and child, respectively (Table 6). The child population of this region is more exposed to the health threat compared to the adults due to their relatively lighter body weight, similar to the observations of other studies in India and China (Narsimha and Rajitha 2018; Zhang et al. 2018; Karunanidhi et al. 2020b). Total Health Index (THQI) values (> 1) were influenced mainly by HQ_fluoride_ and they indicate that 53% of the groundwater samples could be avoided for drinking as they are unsafe or pose high risk for men, women, and children (USEPA 2019). The samples beyond the allowable limit of HQ are located at the wells of northern (*n* = 3) lake margin and southern (*n* = 3) lake margin (i.e., Coatetelco village) as well as the wells (*n* = 3) in surroundings of the Miacatlán village (Fig. 8).

The data of national caries survey of 2001 indicated an overall fluorosis prevalence of 27.9% in Mexico with the lowest values in Morelos state (3.2%, the study area) and the highest in Durango state (88.8%, Betancourt-Lineares et al. 2013). Only about 11.2% of 12-year-olds lacked dental fluorosis in the semi-arid Durango state, whereas nearly 96.8% of the same age group did not show dental fluorosis in Morelos state (Betancourt-Lineares et al. 2013). The survey of 2011–2014, however, revealed that the dental fluorosis is increasingly becoming a public health problem in the sub-humid to humid central and southern states. The prevalence level on the same age group school-going children in Morelos increased to 33.7% after a decade with an average 0.28–0.50 teeth/student showing permanent caries (ENCD 2018). The community fluorosis index for the children and adolescents increased from < 0.4 to > 2.5 (Betancourt-Lineares et al. 2013; ENCD 2018). This could be due to increasing concentration of F in the groundwater of this region. Similar to above 1.5 mg/L of F in both lake and groundwater from Coatetelco, Huízar Álvarez et al. (2016) reported up to 1.90 mg/L of F in groundwater and spring water of Tenextepango area of Morelos (about 40 km, east of Coatetelco). Higher fluoride in 50% of the total samples of this study could be from weathering of fluoride-bearing minerals present in the limestone as well as volcanic deposits through the rock-water interaction. Minerals such as fluorite might have contributed fluorine to the lake and groundwater, and it needs further evaluation and research (e.g., Narsimha and Rajitha 2018; Adimalla and Li 2018). However, the sustainable development of water resources and supply of safe drinking water in this region are the immediate necessities in order to protect the consuming population from exposure to chronic fluorosis, and both can be achieved by considering some of the measures, such as (a) regular quality monitoring, (b) generation of maps indicating vulnerable parts of the aquifer and public awareness programs about water-borne diseases and their preventions, and (c) identification of possible sources and measure of prevention through implementation of aquifer recharge technique through the check dam constructions and recharge pits to reduce the concentration levels of pollutants.

## Conclusions

Physicochemical characteristics of 20 water samples from the Lake Coatetelco in the tropical central-south Mexico and groundwater from the surrounding wells collected immediately after the wet season were evaluated to determine the suitability for drinking and irrigation as well as to demarcate the samples that might cause health risk for adults and children from the ingestion of nitrate and fluoride, with an aim to provide a baseline data for comparison and monitoring of water quality in the short and long terms. More specifically:

(i) In the trilinear diagram of Piper, the lake water was grouped as Ca-Mg-HCO_3_ and the groundwater samples represented facies varying between Na-HCO_3_-Cl and Ca-Mg-HCO_3_, indicating the heterogenous watershed lithologies, i.e., limestone and the siliciclastic volcanic deposits as well as alluvium. The Gibbs plots indicated the dominant influence of rock-water interaction on water chemistry. The irrigation return flow possibly enriched Cl in some of the groundwater samples characterized by high Cl/Cl + HCO_3_.
(ii) In the lake water, the physical parameters mostly remain within the WHO recommendations. K in all samples and HCO_3_ in one sample remained above the permissible limits. NO_3_ remained below the permissible limit of WHO (50 mg/L) and Mexican norms (42 mg/L) in all the samples, and fluoride in one sample was above 1.5 mg/L. The drinking water quality index (DWQI), however, grouped them in excellent and good categories. In the groundwater samples, NO_3_ remained below the permissible limit of WHO and Mexican norms but shows influence of synthetic nitrogenous fertilizers in wells near the agricultural fields. Fluoride was above the recommended limit of 1.5 mg/L in 53% samples (*n* = 9) showing the possible risk of fluorosis. DWQI demarcated 94% samples in excellent and good categories for drinking and the sample in poor category has the highest F (8.7 mg/L), TDS (1020 mg/L), and Na (308.7 mg/L).
(iii) In the lake water, the indices of salinity hazard and SAR categorized all as suitable for irrigation. The USSL diagram classified them as C2S1 (moderate salinity hazard) and C2S1 (low sodium hazard). The Doneen classification suggested their suitability for irrigation with > 75% of permeability. IWQI, however, grouped all of them as moderate restriction for irrigation. In the groundwater samples, RSC demarcated about 53% samples as unsuitable and Na% categorized about 24% as doubtful for irrigation. The USSL classification helped to demarcate the 71% samples (12 wells), with low and medium salinity hazards (C1S1 and C2S1) for irrigation without any special measure, and 29% samples (5 wells) with high salinity hazard (C3S1) suitable for only salt-tolerant plants. The Doneen diagram divided these samples based on their permeabilities.
(iv) The estimation of non-carcinogenic risks from nitrate and fluoride ingestion for adult male (74 kg), adult female (68 kg), and a child with average weight of 20 kg through Health Quotients (HQ) indicated absence of any risk from NO_3_. HQ_fluoride_, however, shows risks in the order of child > adult female > adult male. About 33% of lake water samples and 53% of groundwater samples show possible non-carcinogenic risk with respect to adults and children. Higher fluoride in 50% of the total samples of this study could be from weathering of fluoride-bearing minerals in the limestone as well as volcanic deposits through the rock-water interaction. Results of this study indicating F > 1.5 mg/L in both lake and groundwater are in congruence with the data of national caries surveys suggesting almost tenfold increase in fluorosis prevalence from 3.2 to 33.7% on the 12-year-old school-going children in Morelos over a decade between 2001 and 2011–2014.

## Data Availability

Data used in this study are included and the raw data will be provided upon request.
